# New Appliances & Things Medical

**Published:** 1909-06-05

**Authors:** 


### NEW APPLIANCES & THINGS MEDICAL.

[We shall be glad to receive at our Office, 28 & 29 Southampton Street, Strand, London, W.C., from the manufacturers, specimens of all new preparations and appliances.]

### KALZEN.

A. Rowan and Brother, Limehouse, London, E.

We have received preparations of a new disinfectant to which the name "Kalzen" has been given. This substance has been supplied to us in three forms—namely (1) a brownish liquid, (2) a fine powder soluble in water, (3) a fine powder insoluble in water. In addition to these, we have also received a Kalzen soap, which is strongly disinfected and makes a good lather. The antiseptic powder of Kalzen has been submitted to a severe series of bacteriological tests which have proved it to possess a very high bactericidal co-efficient. According to these experiments, it is from 10 to 60 times as effective as pure carbolic acid, according to the organism against which it is tested. In the dilution required it is non-poisonous, and even in more concentrated form it is not sufficiently toxic to be dangerous in the household. It is guaranteed not to burn, stain, or discolour.

